# Changes in cancer incidence and mortality in Australia over the period 1996–2015

**DOI:** 10.1186/s13104-020-05395-6

**Published:** 2020-12-10

**Authors:** Qingwei Luo, Julia Steinberg, Dianne L. O’Connell, Paul B. Grogan, Karen Canfell, Eleonora Feletto

**Affiliations:** 1 Cancer Research Division, Cancer Council NSW, Kings Cross, PO Box 572, Sydney, NSW 1340 Australia; 2 The University of Sydney School of Public Health, Faculty of Medicine and Health, The University of Sydney, Sydney, NSW Australia; 3 School of Medicine and Public Health, University of Newcastle, Newcastle, NSW Australia; 4 Prince of Wales Clinical School, University of New South Wales, Sydney, NSW Australia

**Keywords:** Australia, Cancer incidence, Cancer mortality, Cancer control

## Abstract

**Objective:**

A previous Australian study compared the observed numbers of cancer cases and deaths in 2007 with the expected numbers based on 1987 rates. This study examines the impact of cancer rate changes over the 20-year period 1996–2015, for people aged under 75 years.

**Results:**

The overall age-standardised cancer incidence rate increased from 350.7 in 1995 to 364.4 per 100,000 in 2015. Over the period 1996–2015, there were 29,226 (2.0%) more cases (males: 5940, 0.7%; females: 23,286, 3.7%) than expected numbers based on 1995 rates. Smaller numbers of cases were observed compared to those expected for cancers of the lung for males and colorectum, and cancers with unknown primary. Larger numbers of cases were observed compared to those expected for cancers of the prostate, thyroid and female breast. The overall age-standardised cancer mortality rate decreased from 125.6 in 1995 to 84.3 per 100,000 in 2015. During 1996 to 2015 there were 106,903 (− 20.6%) fewer cancer deaths (males: − 69,007, − 22.6%; females: − 37,896, − 17.9%) than expected based on the 1995 mortality rates. Smaller numbers of deaths were observed compared to those expected for cancers of the lung, colorectum and female breast, and more cancer deaths were observed for liver cancer.

## Introduction

Australia has one of the highest cancer incidence rates in the world [1]. A previous Australian study compared numbers of cancer cases and deaths observed in 2007 with expected numbers based on 1987 rates, with much of the difference likely to be due to the impact of cancer control measures [2]. This prior study showed that over the period 1987–2007, for Australians aged under 75, the number of cancer deaths was 61,000 lower than it would have been based on the 1987 rates [2]. The number of cancer cases, however, was greater than would have been expected based on 1987 rates [2]. It was posited that improvements in prevention, early detection and treatments played an integral role in the mortality reduction. Some of the increased incidence was associated with increased use of medical services, such as the widespread uptake from the early 1990s of prostate specific antigen (PSA) testing to assess prostate cancer risk [2].

There have been significant developments in cancer control in Australia over the past few decades [2]. Tobacco control was one of the most successful, which through reduced prevalence of smoking is estimated to have prevented 78,000 deaths from lung cancer alone between 1956 and 2015 [3, 4]. Additionally, organised early detection is provided through national screening programs for cancers of the cervix, breast and colorectum, which have all experienced reductions in mortality [5]. The continued development of cancer control initiatives based on the most recent evidence is vital. To identify areas that warrant further attention this analysis provides more recent summary measures of changes from 1996 to 2015 in cancer incidence and mortality for Australian people aged under 75 years.

## Main text

### Methods

Population and tabulated data with the numbers of new cancer cases and deaths by sex, 5-year age group and calendar year from 1994 to 2016 for all cancers combined (ICD-10 codes C00–C97, D45, D46, D47.1, D47.3–D47.5) and for 23 individual cancer types were obtained from the Australian Institute of Health and Welfare (AIHW) [6, 7]. The remaining cancer types were grouped as ‘other’. All cancers for children aged 0–14 years were grouped to avoid small numbers. The analyses were restricted to those aged less than 75 years because by default cancer is a more prevalent cause of death for older people who have survived premature mortality from other chronic diseases, data on cause of death and diagnoses are less reliable at older ages, screening programs stop at approximately 74 years of age, and treatment regimens tend to be less aggressive for older patients [2]. Data were smoothed using a three-year moving average to reduce random variation by single year, and 1995 was used as the reference year.

### Statistical analysis

The methods have been described previously [2]. In brief, Joinpoint regression analysis was used to test for significant changes in cancer rates and estimate the average annual percentage change (AAPC) over the period from 1995 to 2015 [8]. We also reported the annual percentage change (APC) over the last period identified by Joinpoint. We estimated the change in numbers of cancer cases and deaths due to changes in cancer rates rather than changes in the population size and ageing. To do this, we first estimated the expected numbers of cancer cases and deaths for the 20-year period 1996–2015 if rates had remained at 1995 levels, by multiplying the sex-age-specific rates in 1995 by the estimated populations in 1996–2015 [2]. We then calculated the differences in the expected cumulative numbers of cases and deaths and the corresponding observed numbers [2]. All rates were age-standardised to the Australian 2001 population. Statistical analyses were performed using Joinpoint 4.7.0.0 [8] and STATA [9].

### Results

#### Cancer incidence

**Fig. 1:**
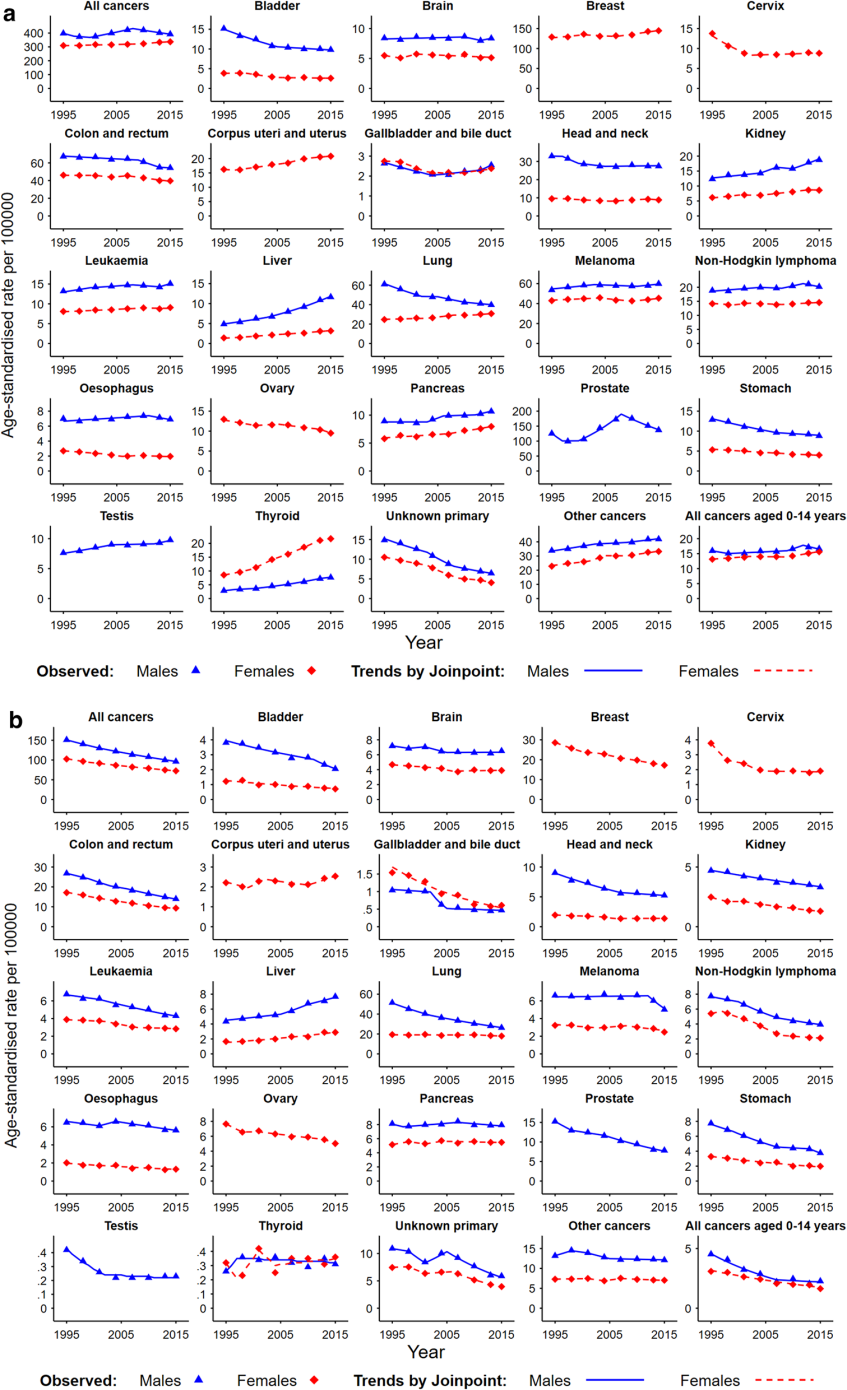
Trends in age-standardised incidence and mortality rates in Australia for 1995–2015 from Joinpoint regression analysis for people aged under 75 years (**a** Incidence; **b** mortality). All rates were age-standardised to the Australian 2001 population. p < 0.05 was considered to indicate statistical significance for the Joinpoint analysis of changes in rates. C26.0 (“malignant neoplasms of the intestinal tract unspecified”) was included for colorectal cancer mortality, as it has been reported that deaths certified as ‘bowel cancer’ tend to be coded as C26.0 [7]

The overall age-standardised incidence rate for all cancers combined increased by 3.9% from 350.7 in 1995 to 364.4 per 100,000 in 2015 (Fig. 1a and Table 1A). Different patterns were observed for males and females, with the incidence rate for all cancers combined for males showing fluctuations over the 20-year period (AAPC: − 0.1, 95% CI − 0.4, 0.2), while a significant increase in the incidence rate for all cancers combined was observed for females (AAPC: 0.5, 95% CI 0.4, 0.5). Notably, in the last period identified by Joinpoint there were opposing changes in cancer incidence rates for males and females, with a decrease observed for males (APC: − 1.4, 95% CI − 1.6, − 1.3 in 2008–2015) and an increase observed for females (APC: 0.9, 95% CI 0.7, 1.0 in 2009–2015) (Table 1A). During 1996–2015, a total of 29,226 (2.0%) more cases (males: 5,940, 0.7%; females: 23,286, 3.7%) were observed compared to the expected numbers based on 1995 rates (Table 2A). For males, compared with expected, the largest differences with smaller numbers of cases observed were for lung (− 23,156, − 23.8%) and colorectal (− 9,092 − 8.5%) cancer, while larger numbers of cases were observed for prostate cancer (29,638, 14.8%) and melanoma (7,208, 8.6%). For females, compared with expected, smaller numbers of cases were observed for cervical cancer (− 7,405, − 34.7%) and cancers with unknown primary (CUP) (− 6,123, − 35.8%), while larger numbers of cases were observed for thyroid (10,993, 83.6%%) and breast (9,952, 4.9%) cancers.

**Table 1:** Changes in age-standardised cancer incidence and mortality rates for people aged under 75 years in Australia over the study period from 1995 to 2015 and the last period identified by Joinpoint analysis (A incidence rate; B mortality rate)

Cancer type	Over the study period from 1995 to 2015						Over the last period identified by Joinpoint					
	Males		Females		Total		Males		Females		Total	
	ASR	AAPC (95%CI)	ASR	AAPC (95%CI)	ASR	AAPC (95%CI)	Period	APC (95%CI)	Period	APC (95%CI)	Period	APC (95%CI)
**(A) Incidence rate (ranked by the total numbers of cases observed in 2015)**												
All cancers aged 0–74 years	397.8–392.6	− 0.1 (− 0.4, 0.2)*	309.9–337.3	0.5 (0.4, 0.5)	350.7–364.4	0.2 (0.1, 0.3)	2008–2015	− 1.4 (− 1.6, − 1.3)	2009–2015	0.9 (0.7, 1.0)	2008–2015	− 0.4 (− 0.5, − 0.3)
Aged 15–74 years												
Prostate (C61)	125.5–137.2	0.5 (− 0.9, 1.8)*			125.5–137.2	0.5 (− 0.9, 1.8)*	2008–2015	− 4.5 (− 5.2, − 3.7)			2008–2015	− 4.5 (− 5.2, − 3.7)
Breast (C50)			128.8–144.6	0.7 (0.4, 0.9)	128.8–144.6	0.7 (0.4, 0.9)			2007–2015	1.4 (1.1, 1.7)	2007–2015	1.4 (1.1, 1.7)
Colon and rectum (C18–C20)	67.4–54.5	− 1.1 (− 1.4, − 0.8)	46.2–39.7	− 0.8 (− 1.4, − 0.2)	56.5–46.9	− 1.0 (− 1.2, − 0.8)	2013–2015	− 0.6 (− 3.0, 1.7)*	2007–2015	− 1.8 (− 2.1, − 1.6)	2008–2015	− 2.2 (− 2.6, − 1.8)
Melanoma (C43)	53.7–59.9	0.4 (0.2, 0.7)	42.9–45.4	0.2 (− 0.1, 0.5)*	48.2–52.5	0.3 (0.1, 0.6)	2011–2015	0.9 (0.0, 1.9)	2010–2015	1.3 (0.6, 1.9)	2011–2015	1.3 (0.4, 2.2)
Lung (C33–C34)	61.0–39.7	− 2.1 (− 2.4, − 1.9)	24.6–30.8	1.1 (0.7, 1.5)	42.1–35.2	− 0.9 (− 1.1, − 0.7)	2010–2015	− 1.2 (− 1.5, − 1.0)	2012–2015	1.5 (0.3, 2.8)	2011–2015	− 0.2 (− 0.5, 0.2)*
Head and neck (C00–C14, C30–C32)	32.9–27.4	− 0.9 (− 1.6, − 0.1)	9.5–8.8	− 0.5 (− 0.9, − 0.1)	21.0–18.0	− 0.8 (− 1.0, − 0.5)	2005–2015	0.1 (− 0.1, 0.4)*	2013–2015	− 2.4 (− 5.8, 1.2)*	2004–2015	0.3 (0.0, 0.6)
Non-Hodgkin lymphoma (C82–C85)	18.9–20.2	0.4 (− 0.1, 0.9)*	14.1–14.6	0.1 (− 0.3, 0.6)*	16.5–17.4	0.4 (0.3, 0.5)	2012–2015	− 1.7 (− 3.2, − 0.2)	2008–2015	0.7 (0.5, 1.0)	1995–2015	0.4 (0.3, 0.5)
Kidney (C64)	12.4–18.8	2.0 (0.5, 3.6)	6.2–8.6	1.8 (1.2, 2.5)	9.2–13.6	1.9 (1.8, 2.1)	2010–2015	3.8 (2.3, 5.4)	2013–2015	− 0.6 (− 4.2, 3.1)*	1995–2015	1.9 (1.8, 2.1)
Leukaemia (C91–C95)	13.2–15.1	0.8 (0.4, 1.1)	8.1–9.1	0.5 (0.2, 0.8)	10.6–12.1	0.7 (0.4, 1.1)	2013–2015	2.7 (0.6, 5.0)	2010–2015	− 0.2 (− 1.1, 0.7)*	2013–2015	2.4 (0.5, 4.2)
Thyroid (C73)	2.9–7.6	5.1 (4.2, 6.0)	8.6–21.7	4.8 (4.5, 5.1)	5.7–14.7	4.9 (4.6, 5.2)	2013–2015	2.9 (− 1.4, 7.4)*	2013–2015	1.0 (− 0.9, 3.0)*	2013–2015	1.7 (0.2, 3.3)
Corpus uteri and uterus (C54–C55)			16.3–20.8	1.3 (0.9, 1.7)	16.3–20.8	1.3 (0.9, 1.7)			2010–2015	0.9 (0.5, 1.3)	2010–2015	0.9 (0.5, 1.3)
Unknown primary (C77–C80, C97)	14.8–6.4	− 4.2 (− 4.7, − 3.8)	10.5–4.1	− 4.7 (− 5.2, − 4.2)	12.6–5.2	− 4.3 (− 4.7, − 4.0)	2008–2015	− 3.4 (− 4.0, − 2.9)	2013–2015	− 6.9 (− 9.8, − 4.0)	2008–2015	− 3.7 (− 4.1, − 3.3)
Pancreas (C25)	8.9–10.7	1.0 (0.4, 1.6)	5.8–8.0	1.5 (1.2, 1.8)	7.3–9.3	1.1 (0.9, 1.4)	2012–2015	2.2 (0.6, 3.8)	2007–2015	2.2 (1.6, 2.9)	2001–2015	1.5 (1.4, 1.7)
Stomach (C16)	12.9–8.8	− 1.9 (− 2.1, − 1.7)	5.3–4.0	− 1.6 (− 1.8, − 1.5)	9.0–6.4	− 1.8 (− 2.0, − 1.6)	2007–2015	− 0.9 (− 1.3, − 0.5)	1995–2015	− 1.6 (− 1.8, − 1.5)	2007–2015	− 1.0 (− 1.4, − 0.7)
Bladder (C67)	15.2–9.8	− 2.1 (− 2.4, − 1.9)	3.8–2.6	− 1.9 (− 2.9, − 1.0)	9.3–6.1	− 2.0 (− 2.5, − 1.5)	2005–2015	− 0.8 (− 1.1, − 0.5)	2005–2015	− 0.7 (− 1.3, 0.0)*	2004–2015	− 0.8 (− 1.0, − 0.6)
Brain (C71)	8.4–8.4	0.1 (− 0.6, 0.7)*	5.5–5.1	− 0.3 (− 1.8, 1.2)*	6.9–6.7	− 0.2 (− 0.5, 0.2)*	2013–2015	2.1 (− 1.8, 6.1)*	2001–2015	− 0.7 (− 1.1, − 0.3)	2010–2015	− 1.3 (− 2.6, − 0.1)
Ovary (C56)			13.0–9.5	− 1.5 (− 2.0, − 0.9)	13.0–9.5	− 1.5 (− 2.0, − 0.9)			2013–2015	− 4.5 (− 7.9, − 1.1)	2013–2015	− 4.5 (− 7.9, − 1.1)
Liver (C22)	4.9–11.6	4.5 (4.3, 4.7)	1.4–3.2	4.5 (4.0, 5.1)	3.1–7.3	4.6 (4.5, 4.7)	2003–2015	5.1 (4.8, 5.3)	2004–2015	3.8 (3.1, 4.4)	1995–2015	4.6 (4.5, 4.7)
Oesophagus (C15)	7.0–6.9	0.2 (− 0.2, 0.5)*	2.7–2.0	− 1.7 (− 2.8, − 0.5)	4.8–4.4	− 0.4 (− 0.9, 0.2)*	2011–2015	− 1.8 (− 3.2, − 0.4)	2009–2015	− 1.2 (− 2.1, − 0.2)	2009–2015	− 1.4 (− 2.0, − 0.9)
Cervix (C53)			13.8–8.8	− 1.9 (− 2.2, − 1.7)	13.8–8.8	− 1.9 (− 2.2, − 1.7)			2002–2015	0.5 (0.3, 0.8)	2002–2015	0.5 (0.3, 0.8)
Testis (C62)	7.6–9.8	1.3 (1.0, 1.6)			7.6–9.8	1.3 (1.0, 1.6)	2012–2015	2.0 (0.4, 3.7)			2012–2015	2.0 (0.4, 3.7)
Gallbladder and bile duct (C23–C24)	2.6–2.5	− 0.3 (− 1.0, 0.5)*	2.7–2.4	− 0.7 (− 1.5, 0.0)*	2.7–2.5	− 0.5 (− 1.3, 0.2)*	2013–2015	5.3 (− 0.7, 11.8)*	2008–2015	1.6 (0.9, 2.3)	2009–2015	2.2 (1.4, 3.0)
Other cancers	33.9–41.9	1.2 (0.9, 1.4)	22.8–33.2	1.8 (1.3, 2.4)	28.3–37.5	1.4 (1.0, 1.8)	2009–2015	1.1 (0.7, 1.5)	2009–2015	1.8 (1.3, 2.2)	2013–2015	0.5 (− 1.4, 2.4)*
All cancers aged 0–14 years	16.0–16.7	0.2 (− 0.5, 0.9)*	13.1–15.6	0.9 (0.6, 1.2)	14.6–16.1	0.6 (− 0.1, 1.2)*	2012–2015	− 2.5 (− 4.4, − 0.5)	2009–2015	2.1 (1.5, 2.7)	2012–2015	− 0.5 (− 2.6, 1.5)*
**(B) Mortality rate (ranked by the total numbers of deaths observed in 2015)**												
All cancers aged 0–74 years	151.1–96.3	− 2.2 (− 2.3, − 2.2)	102.6–72.6	− 1.7 (− 1.8, − 1.7)	125.6–84.3	− 2.0 (− 2.0, − 1.9)	2001–2015	− 2.1 (− 2.2, − 2.1)	1999–2015	− 1.6 (− 1.7, − 1.6)	2000–2015	− 1.9 (− 1.9, − 1.9)
Aged 15–74 years												
Lung (C33–C34)	51.5–26.3	− 3.2 (− 3.3, − 3.2)	19.4–18.0	− 0.3 (− 0.6, − 0.1)	34.8–22.1	− 2.2 (− 2.4, − 2.1)	2002–2015	− 2.9 (− 3.0, − 2.8)	2011–2015	− 1.4 (− 2.8, 0.0)*	2011–2015	− 2.3 (− 2.9, − 1.8)
Colon and rectum (C18–C20, C26.0)	26.8–14.0	− 3.2 (− 3.5, − 3.0)	17.1–9.3	− 3.2 (− 3.4, − 3.0)	21.8–11.6	− 3.2 (− 3.3, − 3.1)	2006–2015	− 3.5 (− 3.7, − 3.3)	1995–2015	− 3.2 (− 3.4, − 3.0)	1995–2015	− 3.2 (− 3.3, − 3.1)
Breast (C50)			28.6–17.2	− 2.5 (− 2.9, − 2.1)	28.6–17.2	− 2.5 (− 2.9, − 2.1)			2003–2015	− 2.4 (− 2.6, − 2.2)	2003–2015	− 2.4 (− 2.6, − 2.2)
Unknown primary (C77–C80, C97)	10.9–5.9	− 3.2 (− 4.2, − 2.3)	7.4–4.0	− 3.3 (− 3.9, − 2.6)	9.1–4.9	− 3.2 (− 3.9, − 2.5)	2005–2015	− 5.9 (− 6.4, − 5.4)	2006–2015	− 5.9 (− 6.4, − 5.5)	2005–2015	− 5.7 (− 6.1, − 5.3)
Pancreas (C25)	8.1–7.9	− 0.2 (− 0.5, 0.2)*	5.2–5.5	0.3 (− 0.9, 1.5)*	6.6–6.7	0.1 (0.0, 0.3)*	2007–2015	− 0.8 (− 1.2, − 0.4)	2004–2015	− 0.3 (− 0.7, 0.1)*	2006–2015	− 0.4 (− 0.7, − 0.2)
Brain (C71)	7.2–6.5	− 0.6 (− 1.3, 0.1)*	4.7–3.9	− 0.9 (− 1.5, − 0.2)	5.9–5.2	− 0.7 (− 1.1, − 0.3)	2005–2015	0.1 (− 0.3, 0.4)*	2006–2015	0.3 (− 0.1, 0.8)*	2006–2015	0.3 (0.0, 0.5)*
Prostate (C61)	15.3–7.9	− 3.4 (− 3.7, − 3.1)			15.3–7.9	− 3.4 (− 3.7, − 3.1)	2004–2015	− 3.8 (− 4.1, − 3.5)			2004–2015	− 3.8 (− 4.1, − 3.5)
Melanoma (C43)	6.6–5.0	− 1.1 (− 1.7, − 0.6)	3.2–2.5	− 1.1 (− 2.1, − 0.2)	4.9–3.7	− 1.1 (− 1.5, − 0.7)	2012–2015	− 8.0 (− 11.2, − 4.7)	2008–2015	− 2.6 (− 4.0, − 1.1)	2012–2015	− 6.9 (− 9.6, − 4.0)
Non-Hodgkin lymphoma (C82–C85)	7.7–4.0	− 3.3 (− 3.7, − 2.9)	5.4–2.1	− 4.6 (− 5.0, − 4.1)	6.5–3.0	− 3.8 (− 4.1, − 3.5)	2007–2015	− 2.6 (− 3.2, − 2.0)	2008–2015	− 2.2 (− 3.0, − 1.4)	2008–2015	− 2.3 (− 2.8, − 1.9)
Leukaemia (C91–C95)	6.8–4.3	− 2.2 (− 2.5, − 1.9)	3.9–2.8	− 1.5 (− 1.7, − 1.2)	5.3–3.6	− 1.9 (− 2.4, − 1.5)	2001–2015	− 2.6 (− 2.9, − 2.3)	2008–2015	− 0.6 (− 1.0, − 0.1)	2005–2015	− 2.1 (− 2.4, − 1.8)
Liver (C22)	4.3–7.7	2.6 (2.2, 3.0)	1.7–2.9	3.3 (2.9, 3.6)	3.0–5.3	2.9 (2.6, 3.1)	2010–2015	2.7 (1.9, 3.5)	1995–2015	3.3 (2.9, 3.6)	2003–2015	3.5 (3.3, 3.7)
Head and neck (C00–C14, C30–C32)	9.0–5.2	− 2.7 (− 3.0, − 2.3)	2.0–1.4	− 1.7 (− 3.0, − 0.4)	5.4–3.3	− 2.5 (− 3.1, − 1.9)	2007–2015	− 1.1 (− 1.8, − 0.4)	2006–2015	0.2 (− 0.7, 1.1)*	2006–2015	− 0.9 (− 1.4, − 0.5)
Oesophagus (C15)	6.5–5.6	− 0.8 (− 1.4, − 0.2)	2.0–1.3	− 2.1 (− 2.4, − 1.8)	4.2–3.4	− 1.1 (− 1.6, − 0.6)	2004–2015	− 1.5 (− 1.8, − 1.2)	1995–2015	− 2.1 (− 2.4, − 1.8)	2004–2015	− 1.7 (− 2.0, − 1.3)
Stomach (C16)	7.7–3.8	− 3.5 (− 4.1, − 2.9)	3.3–2.0	− 2.8 (− 3.1, − 2.4)	5.4–2.8	− 3.2 (− 3.9, − 2.5)	2013–2015	− 6.9 (− 11.8, − 1.7)	1995–2015	− 2.8 (− 3.1, − 2.4)	2013–2015	− 5.2 (− 10.6, 0.6)*
Ovary (C56)			7.7–5.0	− 2.1 (− 2.8, − 1.3)	7.7–5.0	− 2.1 (− 2.8, − 1.3)			2013–2015	− 5.0 (− 9.7, − 0.1)	2013–2015	− 5.0 (− 9.7, − 0.1)
Kidney (C64)	4.7–3.3	− 1.7 (− 1.9, − 1.5)	2.5–1.3	− 3.2 (− 4.1, − 2.3)	3.6–2.3	− 2.1 (− 2.2, − 2.0)	1995–2015	− 1.7 (− 1.9, − 1.5)	2001–2015	− 3.5 (− 3.8, − 3.1)	1995–2015	− 2.1 (− 2.2, − 2.0)
Bladder (C67)	3.8–2.1	− 3.2 (− 3.9, − 2.5)	1.2–0.7	− 2.7 (− 3.1, − 2.3)	2.5–1.4	− 3.0 (− 3.7, − 2.4)	2011–2015	− 6.5 (− 9.7, − 3.1)	1995–2015	− 2.7 (− 3.1, − 2.3)	2011–2015	− 5.8 (− 8.9, − 2.7)
Corpus uteri and uterus (C54–C55)			2.2–2.5	0.6 (− 0.4, 1.7)*	2.2–2.5	0.6 (− 0.4, 1.7)*			2010–2015	4.0 (2.6, 5.4)	2010–2015	4.0 (2.6, 5.4)
Cervix (C53)			3.8–1.9	− 3.5 (− 4.5, − 2.4)	3.8–1.9	− 3.5 (− 4.5, − 2.4)			2004–2015	− 0.3 (− 0.7, 0.2)*	2004–2015	− 0.3 (− 0.7, 0.2)*
Gallbladder and bile duct (C23–C24)	1.0–0.5	− 4.0 (− 5.9, − 2.1)	1.5–0.6	− 5.6 (− 6.2, − 5.1)	1.3–0.5	− 4.8 (− 7.1, − 2.5)	2005–2015	− 1.0 (− 2.4, 0.4)*	1995–2015	− 5.6 (− 6.2, − 5.1)	2005–2015	− 3.4 (− 5.0, − 1.7)
Thyroid (C73)	0.3–0.3	1.0 (− 1.2, 3.4)*	0.3–0.4	0.4 (− 3.8, 4.9)*	0.3–0.3	0.8 (− 1.2, 2.8)*	1997–2015	− 0.5 (− 1.1, 0.1)*	2004–2015	1.3 (− 0.3, 2.8)*	2004–2015	0.5 (− 0.2, 1.1)*
Testis (C62)	0.4–0.2	− 3.4 (− 4.3, − 2.4)			0.4–0.2	− 3.4 (− 4.3, − 2.4)	2002–2015	− 0.9 (− 2.1, 0.3)*			2002–2015	− 0.9 (− 2.1, 0.3)*
Other cancers	13.2–12.1	− 0.4 (− 0.8, 0.0)*	7.3–7.0	− 0.2 (− 0.8, 0.4)*	10.1–9.5	− 0.3 (− 0.7, 0.1)*	2005–2015	− 0.3 (− 0.5, 0.0)	2007–2015	− 0.8 (− 1.1, − 0.5)	2004–2015	− 0.2 (− 0.4, − 0.1)
All cancers aged 0–14 years	4.5–2.3	− 3.6 (− 4.5, − 2.7)	3.1–1.6	− 2.9 (− 3.4, − 2.5)	3.8–2.0	− 3.2 (− 3.9, − 2.5)	2007–2015	− 1.0 (− 3.2, 1.2)*	1995–2015	− 2.9 (− 3.4, − 2.5)	2008–2015	− 1.1 (− 3.1, 0.9)*

All cancers combined: ICD-10 codes C00–C97, D45, D46, D47.1, D47.3–D47.5. C26.0 (“malignant neoplasms of the intestinal tract unspecified”) was included in colorectal cancer mortality, as it has been reported that deaths certified as ‘bowel cancer’ tended to be coded to C26.0 [7]

*ASR* age-standardised rate. All rates were age-standardised to the Australian 2001 population, *AAPC* average annual percent change over the whole study period from 1995 to 2015, *APC* annual percent change, *CI* confidence interval

*Change not statistically significant (p value ≥ 0.05) for the Joinpoint analysis of changes in rates

**Table 2:** Changes in cumulative numbers of new cases and deaths in Australia over the period 1996–2015 by sex for people aged under 75 years (A number of new cases; B number of deaths)

Cancer type	Observed in 1996–2015 (O)			Expected in 1996–2015 (E)^a^		Difference (O − E)				% change [100 × (O − E)/E]		
	Male	Female	Total	Male	Female	Male	Total	Female	Total	Male	Female	Total
**(A) Number of new cases (ranked by the total numbers of cases observed in 2015)**												
All cancers aged 0–74 years	811,377	655,832	1,467,209	805,437	632,546	1,437,983	5940	23,286	29,226	0.7	3.7	2.0
Aged 15–74 years												
Prostate (C61)	230,348		230,348	200,710		200,710	29,638		29,638	14.8		14.8
Breast (C50)		214,545	214,545		204,593	204,593		9952	9952		4.9	4.9
Colon and rectum (C18–C20)	98,326	70,456	168,782	107,418	75,022	182,440	− 9092	− 4567	− 13,658	− 8.5	− 6.1	− 7.5
Melanoma (C43)	91,016	69,800	160,816	83,808	67,162	150,970	7208	2638	9846	8.6	3.9	6.5
Lung (C33–C34)	74,067	45,530	119,597	97,223	40,198	137,422	− 23,156	5331	− 17,825	− 23.8	13.3	− 13.0
Head and neck (C00–C14, C30–C32)	44,829	14,176	59,006	52,087	15,269	67,356	− 7257	− 1093	− 8350	− 13.9	− 7.2	− 12.4
Non-Hodgkin lymphoma (C82–C85)	31,421	22,760	54,181	29,527	22,698	52,226	1894	62	1956	6.4	0.3	3.7
Kidney (C64)	24,477	12,162	36,639	19,579	9,894	29,473	4897	2268	7166	25.0	22.9	24.3
Leukaemia (C91–C95)	22,572	13,812	36,384	20,747	12,966	33,713	1825	846	2671	8.8	6.5	7.9
Thyroid (C73)	8092	24,150	32,241	4434	13,157	17,590	3658	10,993	14,651	82.5	83.6	83.3
Corpus uteri and uterus (C54–C55)		30,178	30,178		26,353	26,353		3825	3825		14.5	14.5
Unknown primary (C77–C80, C97)	15,491	10,956	26,447	23,574	17,079	40,652	− 8083	− 6123	− 14,205	− 34.3	− 35.8	− 34.9
Pancreas (C25)	15,207	11,178	26,385	14,170	9461	23,632	1037	1717	2753	7.3	18.1	11.7
Stomach (C16)	16,156	7365	23,520	20,448	8617	29,065	− 4292	− 1253	− 5545	− 21.0	− 14.5	− 19.1
Bladder (C67)	17,543	4921	22,465	24,066	6296	30,362	− 6523	− 1375	− 7897	− 27.1	− 21.8	− 26.0
Brain (C71)	13,115	8604	21,719	13,193	8782	21,976	− 78	− 179	− 257	− 0.6	− 2.0	− 1.2
Ovary (C56)		17,894	17,894		20,716	20,716		− 2822	− 2822		− 13.6	− 13.6
Liver (C22)	12,813	3808	16,621	7703	2282	9985	5110	1526	6636	66.3	66.8	66.5
Oesophagus (C15)	11,232	3534	14,766	11,163	4398	15,561	69	− 863	− 795	0.6	− 19.6	− 5.1
Cervix (C53)		13,959	13,959		21,364	21,364		− 7405	− 7405		− 34.7	− 34.7
Testis (C62)	13,314		13,314	11,516		11,516	1799		1799	15.6		15.6
Gallbladder and bile duct (C23-C24)	3607	3785	7392	4191	4474	8665	− 584	− 689	− 1273	− 13.9	− 15.4	− 14.7
Other	60,980	46,568	107,548	53,126	36,493	89,619	7854	10,075	17,928	14.8	27.6	20.0
All cancers aged 0–14 years	6768	5693	12,461	6754	5270	12,024	15	423	437	0.2	8.0	3.6
**(B) Number of deaths (ranked by the total numbers of deaths observed in 2015)**												
All cancers aged 0–74 years	236,831	174,406	411,236	305,838	212,302	518,140	− 69,007	− 37,896	− 106,903	− 22.6	− 17.9	− 20.6
Aged 15–74 years												
Lung (C33–C34)	55,411	30,826	86,237	81,911	31,698	113,610	− 26,500	− 872	− 27,373	− 32.4	− 2.8	− 24.1
Colon and rectum (C18–C20, C26.0)	30,069	19,822	49,892	42,666	27,939	70,604	− 12,596	− 8116	− 20,713	− 29.5	− 29.1	− 29.3
Breast (C50)		34,542	34,542		45,804	45,804		− 11,262	− 11,262		− 24.6	− 24.6
Unknown primary (C77–C80, C97)	13,234	9531	22,765	17,317	12,133	29,450	− 4083	− 2602	− 6685	− 23.6	− 21.4	− 22.7
Pancreas (C25)	12,716	8991	21,707	12,899	8448	21,348	− 183	542	359	− 1.4	6.4	1.7
Brain (C71)	10,302	6538	16,841	11,293	7518	18,811	− 990	− 980	− 1970	− 8.8	− 13.0	− 10.5
Prostate (C61)	16,596		16,596	23,916		23,916	− 7320		− 7320	− 30.6		− 30.6
Melanoma (C43)	9985	4801	14,786	10,389	5116	15,505	− 404	− 315	− 719	− 3.9	− 6.2	− 4.6
Non–Hodgkin lymphoma (C82–C85)	8468	5518	13,987	12,111	8775	20,886	− 3643	− 3256	− 6899	− 30.1	− 37.1	− 33.0
Leukaemia (C91–C95)	8449	5252	13,701	10,586	6243	16,830	− 2137	− 991	− 3129	− 20.2	− 15.9	− 18.6
Liver (C22)	9402	3614	13,015	6886	2699	9585	2516	915	3431	36.5	33.9	35.8
Head and neck (C00–C14, C30–C32)	10,001	2505	12,507	14,439	3180	17,619	− 4438	− 674	− 5112	− 30.7	− 21.2	− 29.0
Oesophagus (C15)	9799	2542	12,340	10,301	3299	13,600	− 502	− 757	− 1260	− 4.9	− 23.0	− 9.3
Stomach (C16)	8093	3924	12,016	12,189	5323	17,511	− 4096	− 1399	− 5495	− 33.6	− 26.3	− 31.4
Ovary (C56)		9899	9899		12,361	12,361		− 2462	− 2462		− 19.9	− 19.9
Kidney (C64)	6202	2846	9048	7478	4045	11,523	− 1276	− 1199	− 2475	− 17.1	− 29.6	− 21.5
Bladder (C67)	4668	1545	6213	6016	1993	8009	− 1348	− 448	− 1796	− 22.4	− 22.5	− 22.4
Corpus uteri and uterus (C54–C55)		3659	3659		3635	3635		24	24		0.7	0.7
Cervix (C53)		3365	3365		5975	5975		− 2610	− 2610		− 43.7	− 43.7
Gallbladder and bile duct (C23–C24)	1058	1535	2593	1659	2528	4187	− 600	− 993	− 1594	− 36.2	− 39.3	− 38.1
Thyroid (C73)	527	523	1051	406	516	922	121	8	129	29.9	1.5	14.0
Testis (C62)	384		384	642		642	− 258		− 258	− 40.2		− 40.2
Other	20,275	11,702	31,978	20,832	11,838	32,670	− 557	− 135	− 693	− 2.7	− 1.1	− 2.1
All cancers aged 0–14 years	1190	926	2116	1901	1237	3138	− 711	− 311	− 1022	− 37.4	− 25.1	− 32.6

All cancers combined: ICD-10 codes C00–C97, D45, D46, D47.1, D47.3–D47.5, C26.0 (“malignant neoplasms of the intestinal tract unspecified”) was included in colorectal cancer mortality, as it has been reported that deaths certified as ‘bowel cancer’ tended to be coded to C26.0 [7]

^a^The expected total number of new cases or deaths in 1996–2015 was calculated by multiplying the smoothed sex-age-specific incidence or mortality rate in 1995 by the estimated populations for 1996–2015

#### Cancer mortality

The age-standardised mortality rate for all cancers combined decreased by 32.9% from 125.6 in 1995 to 84.3 per 100,000 in 2015 (Fig. 1b and Table 1B). Significant decreases in mortality rates were observed for both males (AAPC: − 2.2, 95% CI − 2.3, − 2.2) and females (AAPC: − 1.7, 95% CI − 1.8, − 1.7). Mortality rates for most cancer types, except liver cancer, decreased throughout the 20-year period. There were only negligible changes in mortality rates for cancers of the pancreas, thyroid, corpus uteri and uterus over the whole study period 1995–2015. However, a significant increase in mortality rates for cancer of corpus uteri and uterus in the last period from 2010 to 2015 was identified by Joinpoint (Table 1B). During 1996–2015 there were 106,903 fewer cancer deaths (males: − 69,007, − 22.6%; females: − 37,896, − 17.9%) than expected based on the 1995 mortality rates (Table 2B). For males, compared with expected, the largest differences were for lung (− 26,500, − 32.4%) and colorectal (− 12,596, − 29.5%) cancers. For females compared with expected, the largest differences were for breast (− 11,262, − 24.6%) and colorectal (− 8,116, − 29.1%) cancers. Liver cancer was the only cancer with a significantly larger number of deaths compared to that expected (3.431, 35.8%) (Table 2B).

### Discussion

Our results indicated that the overall incidence rate for all cancers for Australians aged under 75 years was relatively stable from 1995 to 2015, while the mortality rate for all cancers declined by 32.9% over the same period. Over the period 1996–2015, the number of cancer cases was 2% higher and the number of cancer deaths was 20.6% lower compared to those expected based on 1995 rates. The patterns observed in the current study were somewhat consistent with those reported for 1987–2007 [2]. However, there was a greater decrease in the cancer mortality rate and a much smaller increase in the cancer incidence rate [2]. These differences are likely to be attributable to multiple factors including risk factor control, changes in diagnostic practices and cancer screening activities, as well as improvements in cancer treatments, which were highlighted in the previous study [2]. Broadly, Australia has seen a variety of improvements in cancer treatment and the development of tailored and targeted therapies which have led to improvements in cancer outcomes [10]. We identified different patterns in cancer incidence for males and females, with the incidence rate for males being relatively stable while the rate for females showed an increase. These were largely driven by lung cancer incidence due to differences in sex-specific smoking behaviours in the population [3, 11].

For several cancer types that have an established association with tobacco smoking, including cancers of the lung for males, colorectum, head and neck, stomach, bladder, ovary, oesophagus for females and cervix [12], significant decreases in both cancer incidence and mortality rates have resulted in smaller numbers of new cancer cases and cancer deaths being observed compared to those expected based on rates 20 years ago are likely attributable to Australia’s successful tobacco control interventions [3, 4]. However, an increase in the incidence of lung cancer was observed for females, and for lung cancer alone the observed numbers of cases and deaths in 2015 were still substantial, highlighting that continued efforts in tobacco control and the development of effective lung cancer screening and treatments remain significant public health priorities [3]. The first enquiry into the possibility of a national lung cancer screening program is currently progressing in Australia [13].

In the case of colorectal and cervical cancers, organised screening has played a major role in reducing the incidence and mortality rates. Australia’s National Cervical Screening Program (NCSP) was implemented in 1991, the reductions in cervical cancer incidence and mortality rates observed in this study can be mainly attributed to the NCSP. However, a previous study reported that the cervical cancer incidence rate appears to have plateaued during the late 2000s following the levelling off of screening participation rates, and a new screening technology and revised protocol were introduced in 2017 [14]. In addition, the national human papillomavirus (HPV) vaccination program was introduced in April 2007 [15], and over time this will be expected to further reduce the incidence rate of cervical cancer [16]. While the National Bowel Cancer Screening Program (NBCSP) has been phased in from 2006, reaching full implementation from 2020 [5], opportunistic screening occurred prior to its launch and continues, so decreases in colorectal cancer incidence and mortality rates are likely to be attributable to both organised and opportunistic screening [17, 18]. However, in contrast to the overall decrease in the incidence and mortality rates for colorectal cancer, a previous study reported increases in the incidence rates for people aged under 50 years [19]. Further research focusing on the increase in the colorectal cancer incidence rate for young people is warranted.

The decreases in the incidence and mortality rates for CUP could potentially be explained by continuous improvements in diagnostic techniques which may have resulted in these cases being correctly classified as a known primary cancer type [20]. Consequently, this could be a potential factor contributing to the increases in the numbers of new diagnoses of other cancer types compared with expected. Reductions in mortality for cancer types commonly thought to be responsible for CUP, including lung cancer, are likely to have also contributed to the smaller than expected number of deaths from CUP [21].

Increasing or relatively stable incidence rates but decreasing mortality rates were observed for some cancer types, including cancers of the prostate, breast, kidney and melanoma. Although prostate cancer and melanoma are often detected early and treated successfully as a result, currently there is no organised population screening for either as the potential harms are considered to outweigh the benefits [22–25]. The decrease in the prostate cancer incidence rate after 2008 [6] is likely to be attributable to clinical guidelines recommending against PSA testing for prostate cancer since 2008 [22, 23]. For breast cancer, despite debate around mammographic screening [26, 27], assessments of different methods of screening for breast cancer estimated a reduction in mortality of 21–28% for women invited to screening [28, 29].

As found in the previous study which considered a time period starting a decade earlier than the current study, there were statistically significant increases in both incidence and mortality rates for liver cancer, resulting in significantly larger numbers of liver cancer cases and deaths occurring compared to those expected based on 1995 rates [2]. Multiple factors may be the cause of these increases. The prevalence of hepatitis B and C has remained high in some migrant communities, and among Aboriginal and Torres Strait Islander Australians, and there is a substantial burden of undiagnosed chronic hepatitis infections in high risk groups [30]. Other risk factors such as obesity, diabetes and alcohol consumption may also be a factor [2]. In addition, the misclassification of other cancers which have metastasised to the liver could be a factor [31]. Future research focusing on the increase in incidence and mortality rates for liver cancer is a major priority. This study also found that the mortality rate for cancer of corpus uteri and uterus increased in the recent period from 2010 to 2015, which was likely caused by increases in obesity, diabetes, insufficient physical activity, and decreasing use of estrogen plus progestin menopausal hormone therapy [32, 33].

In the context of the Covid-19 pandemic, health services have been severely disrupted in a number of countries and in Australia cancer services have been disrupted to some extent [34], including a pause of BreastScreen Australia services in April–May 2020. Furthermore, population-level health-seeking may be impacted. It will be important to ensure that these disruptions are minimised and that appropriate recovery strategies are in place; one means by which this is occurring is the establishment of the Covid-19 and Cancer Global Modelling Consortium, which aims to help countries minimise the impact of disruptions [35].

### Conclusion

We found that the cancer mortality rate in Australia in 2015 was about a third lower than the rate in 1995, with a total of almost 107,000 fewer deaths over the 20-year period 1996–2015 than would have been expected had the mortality rate remained at the 1995 level. This is likely to in part reflect the success of past cancer control efforts, especially tobacco control, cancer screening, and improvements in treatment. Despite the improvements observed, an increase in cancer incidence remains a concern, with the number of new cancer diagnoses being greater than that expected based on 1995 rates. Although a proportion of these are likely to be due to increased detection, these findings imply that continuing commitment to further research and the implementation of evidence-based cancer control measures must remain public health priorities.

### Limitations

The main limitation of this analysis is that the estimated changes in incidence and mortality are likely to be sensitive to the reference year selected for comparison. To reduce the impact of this and account for random variations in cancer rates for a single year, the 1995 rates were estimated using a three-year moving average.

## Data Availability

The datasets generated and/or analysed for the current study are available from Cancer Data in Australia via: https://www.aihw.gov.au/reports/cancer/cancer-data-in-australia/contents/summary.

## Abbreviations

APC: Annual percent change
AAPC: Average annual percentage change
AIHW: Australian Institute of Health and Welfare
CDA: Cancer Data in Australia
CI: Confidence interval
CUP: Cancers with unknown primary
HPV: Human papilloma virus
ICD: International Classification of Diseases
NBCSP: National Bowel Cancer Screening Program
NCSP: National Cervical Cancer Screening Program
NHL: Non-Hodgkin lymphoma
PSA: Prostate specific antigen
